# Bridging Tradition and Modernity: Embracing the Bipaddled Pectoralis Major Myocutaneous Flap for Challenging Oral Cavity Defects in the Free Flap Era

**DOI:** 10.7759/cureus.62341

**Published:** 2024-06-13

**Authors:** Vaishnavi Gattani, Shreya Pawar, Chetan Gupta, Nitin Bhola, Parmarth Sonpal, Palak Agrawal

**Affiliations:** 1 Oral and Maxillofacial Surgery, Sharad Pawar Dental College and Hospital, Wardha, IND

**Keywords:** metastasis, mandibulectomy, oral cancer, myocutaneous, carcinoma

## Abstract

Oral squamous cell carcinoma is a serious global issue, with the prognosis decreasing as the disease severity increases. The implications of this condition are so disastrous that they cause a lot of suffering for the individual. Early diagnosis has proven to improve patients' overall survival and quality of life. Surgery remains the mainstay in treating oral carcinoma. It is aimed at the complete removal of the cancerous lesion along with the management of cervical nodal metastasis. Larger defects call for reconstruction with bulky flaps. In our case, we had a composite defect postresection of the cancerous lesion, which was reconstructed using a bipaddled pectoralis major myocutaneous flap.

## Introduction

One of the most serious global health challenges today is cancer, which includes a wide range of disorders marked by abnormal cell growth that has the capacity to invade various other body parts. There is a wide range of symptoms, and treatment outcomes are influenced by the complex interplay of genetic, environmental, and lifestyle factors. Within the realm of oncology, carcinoma reigns as one of the most prevalent forms of cancer, encompassing a diverse array of malignancies arising from epithelial tissues. Oral cancer is the sixth most common cancer globally in terms of incidence. Oral cancer emerges as a particularly pervasive threat to public health, exerting a profound impact on individuals and communities worldwide [1].

Squamous cell carcinoma (SCC) accounts for over 90% of all oral cancer cases [2,3]. In head and neck oncology, carcinoma of the lower gingivobuccal sulcus poses a significant problem due to its aggressive nature and frequently delayed diagnosis [4]. Carcinoma originating from the lower gingivobuccal sulcus is a unique subtype that makes up a sizable fraction of cases in areas where betel nut and tobacco use is prevalent, especially in South Asia, which includes Bangladesh, Sri Lanka, and India [5]. For instance, with an estimated yearly incidence of 30-40 cases per 100,000 populations, carcinoma of the lower gingivobuccal sulcus is a major health issue in India [6]. Originating at the interface between the buccal and alveolar mucosa, this variation of oral squamous cell carcinoma (OSCC) presents distinct challenges for diagnosis and treatment. The prevalence of late presentation persists most commonly in areas where there is a lack of awareness and facilities of advances in imaging technologies and treatment modalities, which is associated with poor prognosis and few therapeutic options.

In this report, we describe a case of cancer arising from the lower gingivobuccal sulcus, explaining the clinical manifestation, diagnostic implications, treatment options, and prognosis of the patient. There are many treatment modalities according to the site and size of the lesion; it requires a multimodal approach involving a team comprising surgeons, radiation oncologists, and maxillofacial surgeons. Our goal in presenting this case report is to highlight the value of interdisciplinary cooperation, cutting-edge diagnostic techniques, and customized treatment strategies in maximizing the treatment of this unique form of oral cavity carcinoma focusing on the bipaddled pectoralis major myocutaneous flap (PMMC) with our tailored design adaptation, which endeavors to reduce donor-site complications while optimizing the harvested skin paddle's surface area.

## Case presentation

**Figure 1:**
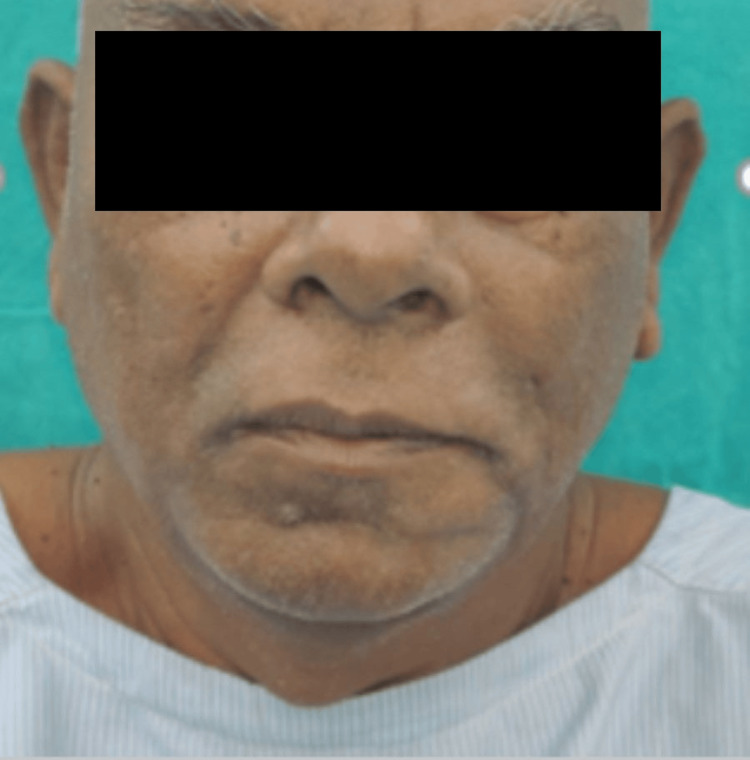
Preoperative frontal view of the patient

A 55-year-old systemically healthy male patient came to the outpatient department of oral and maxillofacial surgery with the chief complaint of a nonhealing ulcer over the left lower side of the jaw, which has been progressing for approximately two to three months (Figure 1). The patient was apparently right two to three months ago when he started noticing a painful nonhealing ulcer over the left region of the lower jaw, which was initially small in size and has gradually progressed to the current size of approximately 4 x 3 cm. He experienced a gradual onset of continuous, dull, and localized pain in that particular region. He also complained of difficulty in mastication and a change in the consistency of saliva from a thin to a thick ropy for approximately 15-20 days. The patient had a history of tobacco consumption five to six times a day for approximately 15 years, placing it in the lower anterior region.

**Figure 2:**
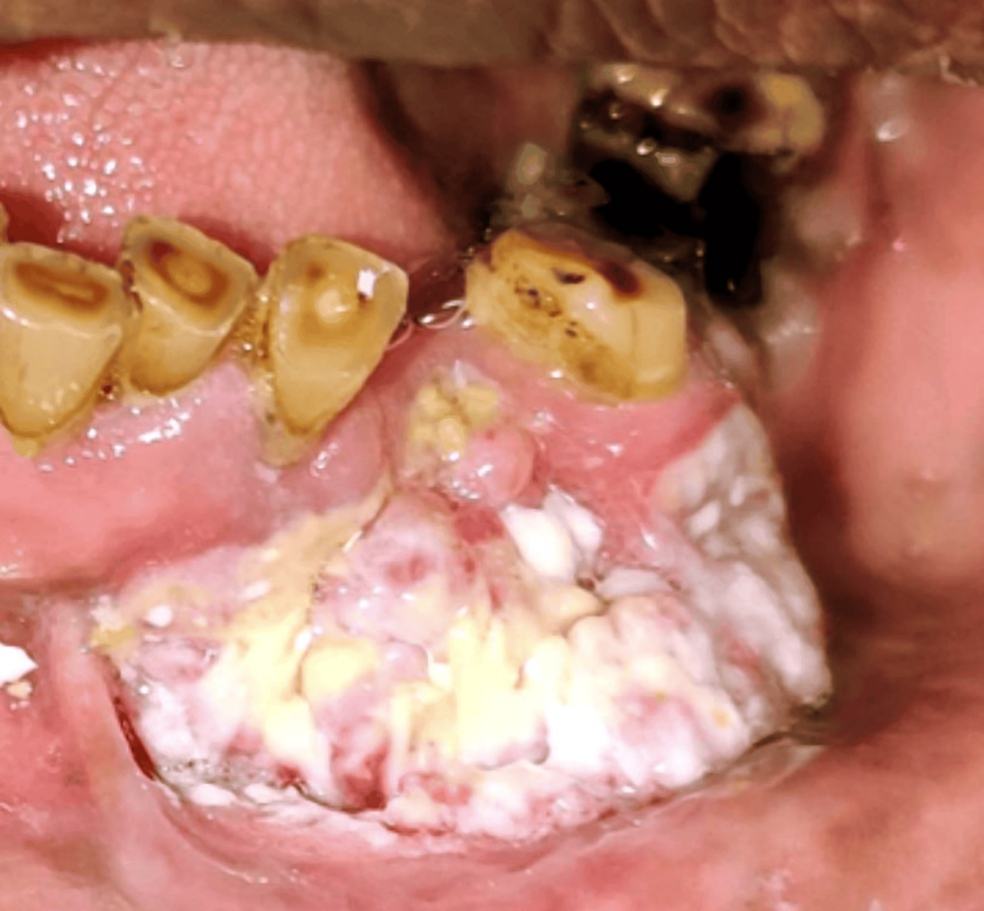
Intraoral figure of the lesion

On examination, the patient had an ulceroproliferative lesion in the lower left gingivobuccal region of size 4 x 3 cm, approximately extending anteroposteriorly from the central incisor of the right side to the first premolar of the left side, superoinferiorly extending from the level of the marginal gingiva to the depth of the lower labial sulcus, which was irregular in shape, pinkish white with everted edges, and ill-defined borders (Figure 2). The lesion was tender on palpation, with soft to firm consistency and induration. The teeth were mobile in the vicinity of the lesion. A single left submandibular lymph node was palpable, measuring 4 x 3 cm, and was roughly oval, nontender, fixed, and firm. Due to these positive clinical findings, our provisional diagnosis was malignancy of the lower left gingivobuccal sulcus with metastasis to the left submandibular lymph node. To confirm our provisional diagnosis, we subjected the patient to an incisional biopsy from the most indurated part of the ulcerative lesion. The report of the biopsy was a well-differentiated SCC.

**Figure 3:**
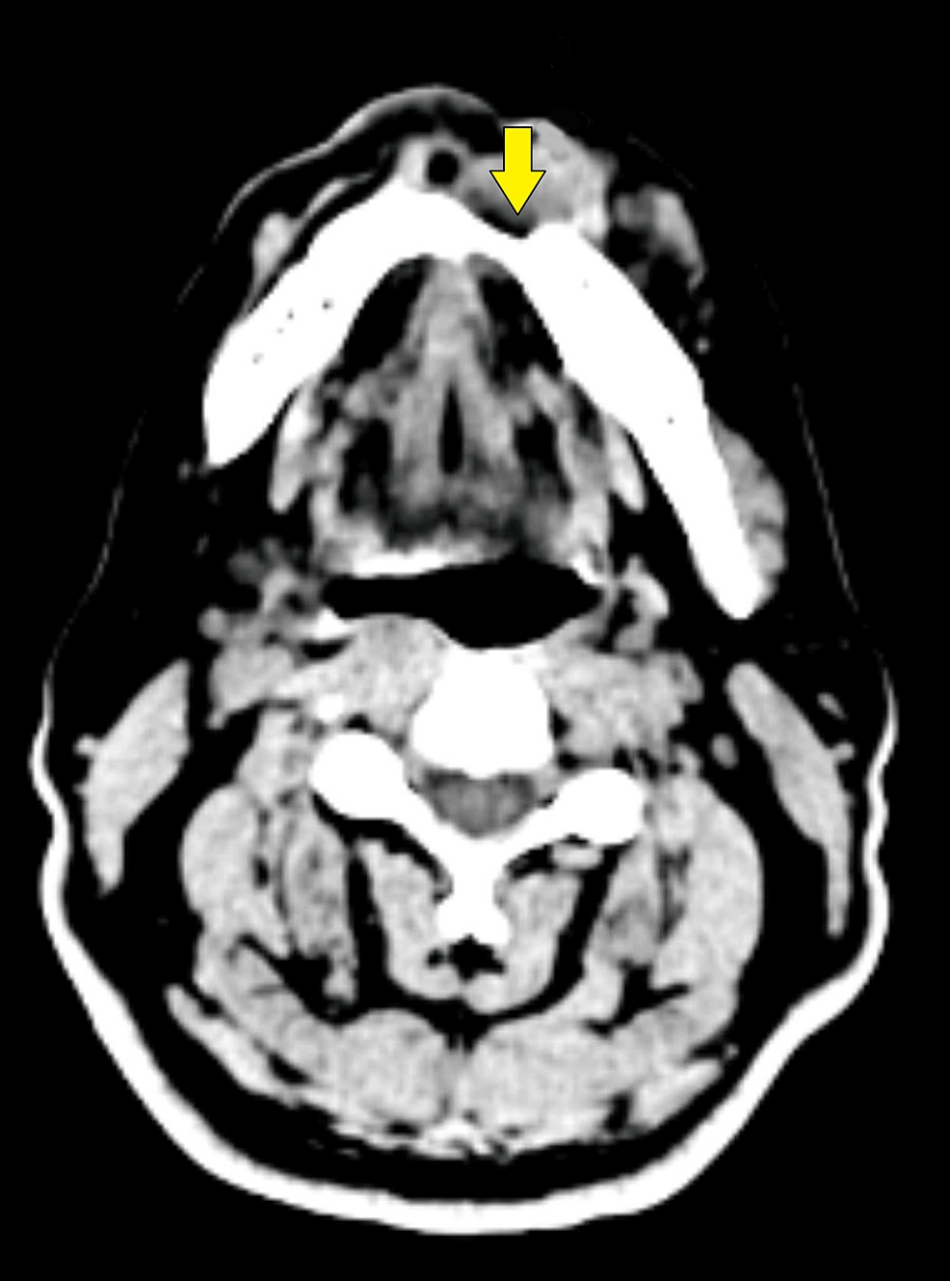
Axial view of the lesion along with contrast (yellow arrow)

**Figure 4:**
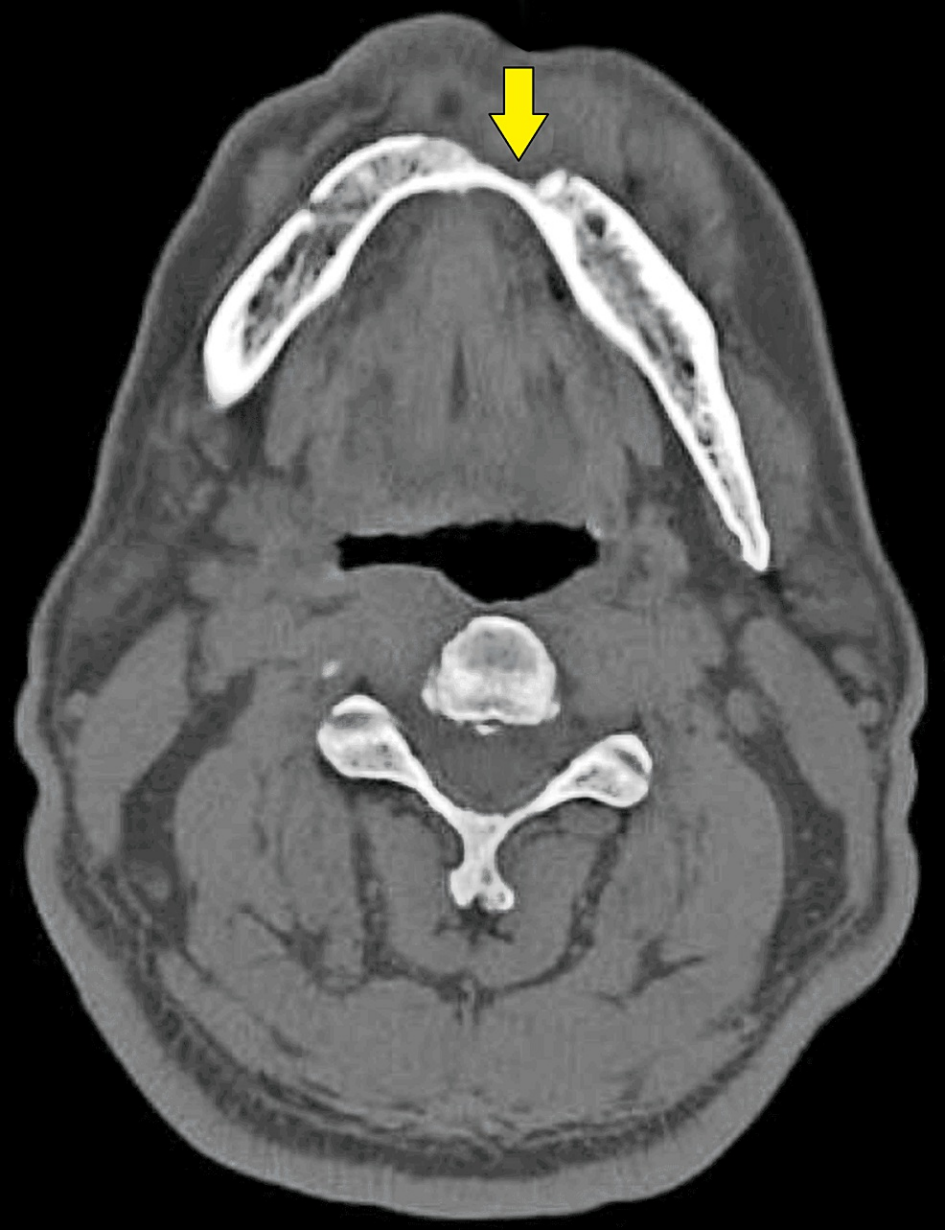
Evidence of bony erosion along with computed tomography scan (yellow arrow)

After confirming our diagnosis, a radiographic examination was done, which consisted of contrast-enhanced computed tomography (CECT) of the head and neck to ascertain the extension of the lesion and high-resolution computed tomography (HRCT) of the thorax for metastatic workup. The CECT report was suggestive of “a heterogeneously enhancing soft tissue density lesion in the lower gingivobuccal sulcus on the left side of symphysis menti causing destruction and erosion of the bone and involving orbicularis oris (Figures 3, 4). The lesion measured 2.3 x 1.7 x 1.6 cm”. The report of the HRCT thorax was suggestive of no metastatic lesion. This case was discussed in a multidisciplinary tumor board meeting at our institute, which was comprised of an oral oncologist, a surgical oncologist, a medical oncologist, a radiation oncologist, and a pathologist. A joint decision was made to proceed with surgical treatment, followed by adjuvant treatment based on the postoperative surgical margin report.

**Figure 5:**
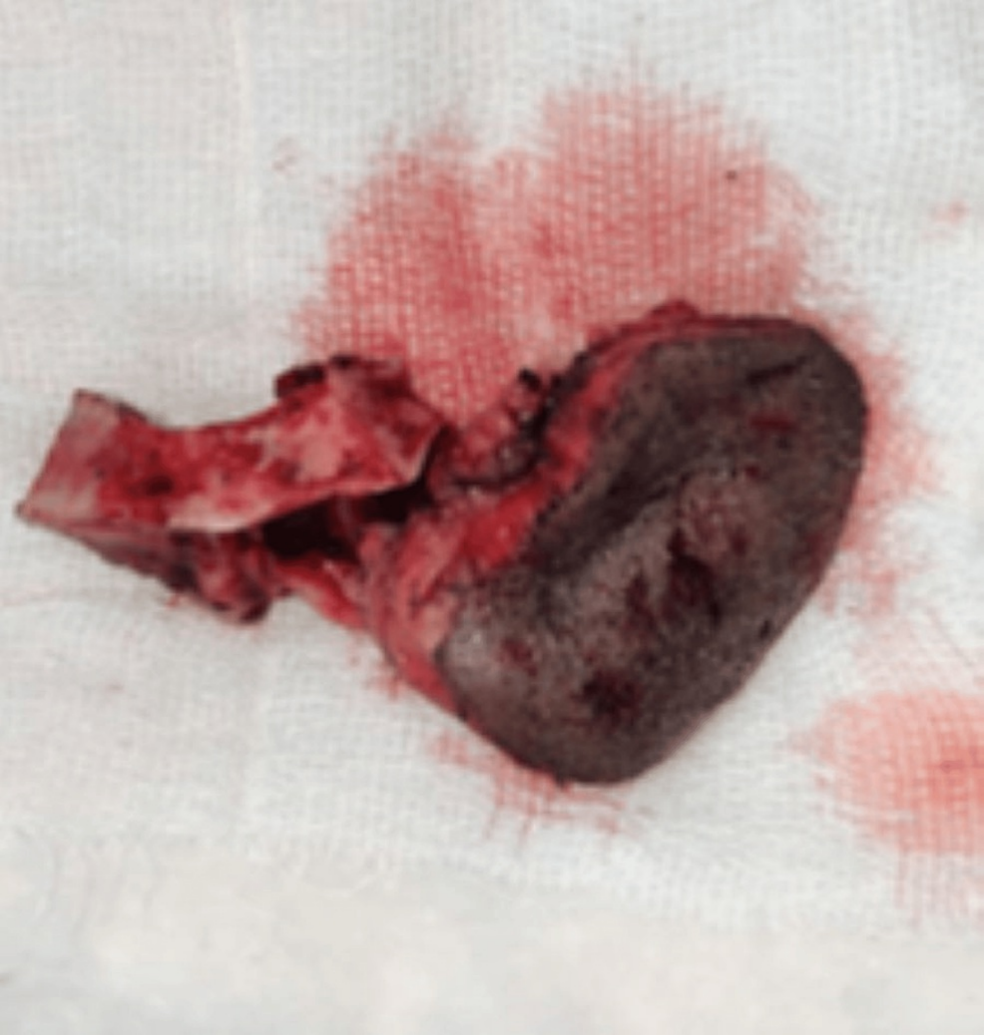
Composite resection of the defect from the left side first molar to the right side lateral incisor

**Figure 6:**
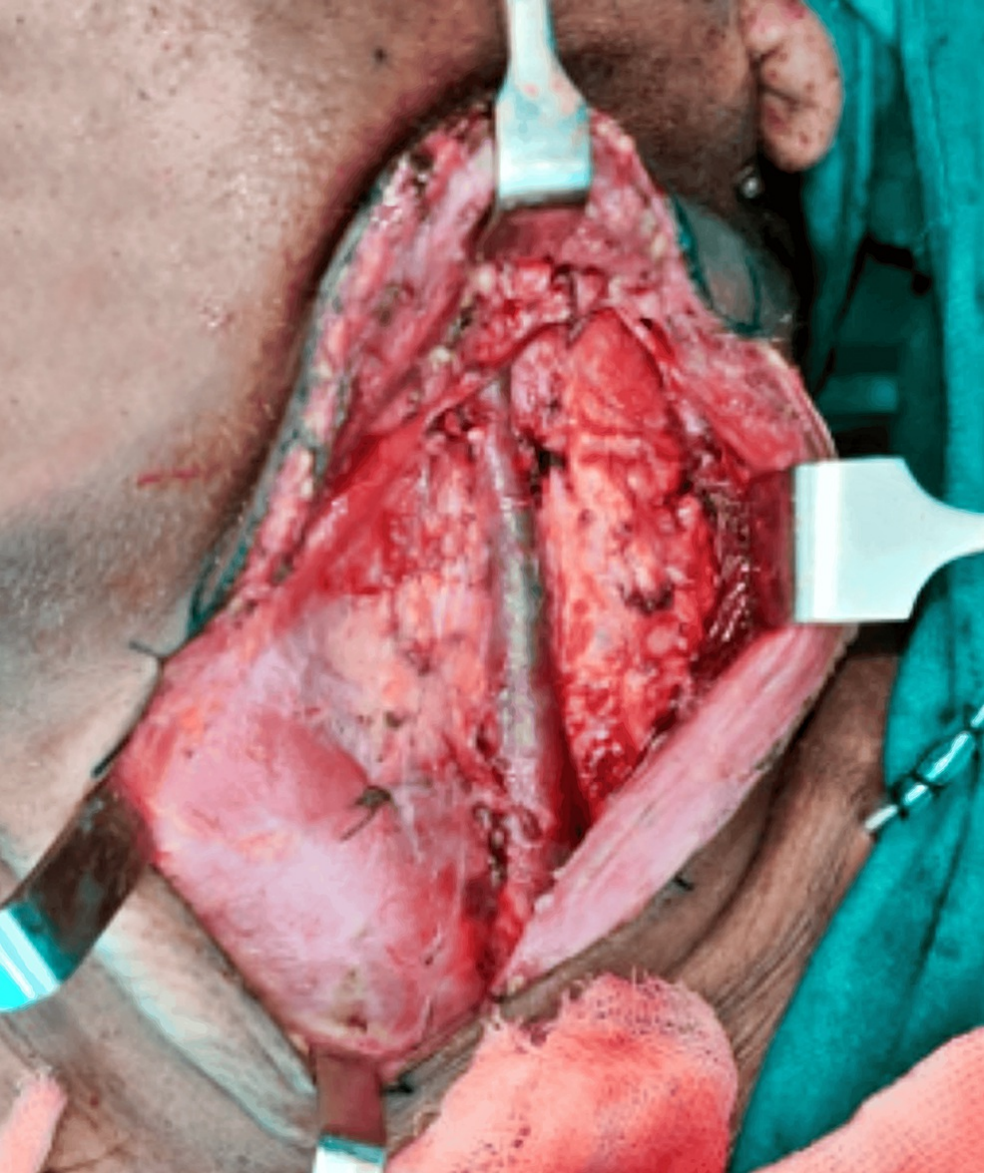
Modified radical neck dissection of the left side

**Figure 7:**
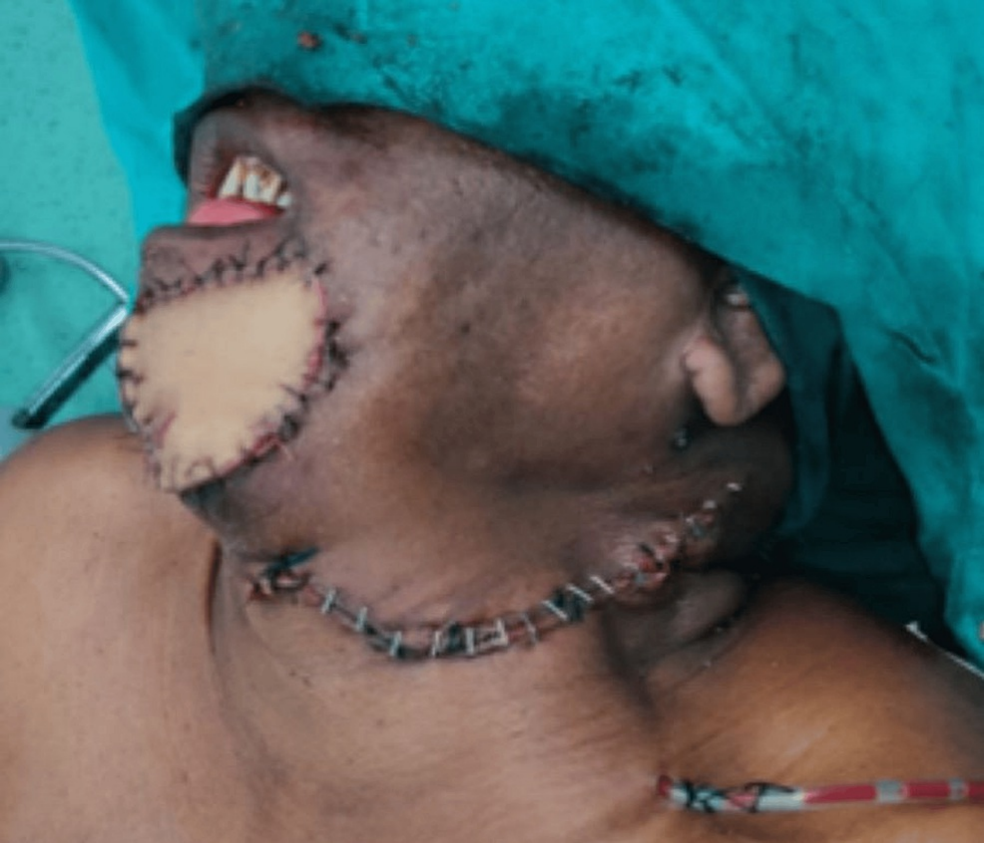
The reconstruction of the defect was done with the PMMC flap PMMC: pectoralis major myocutaneous

**Figure 8:**
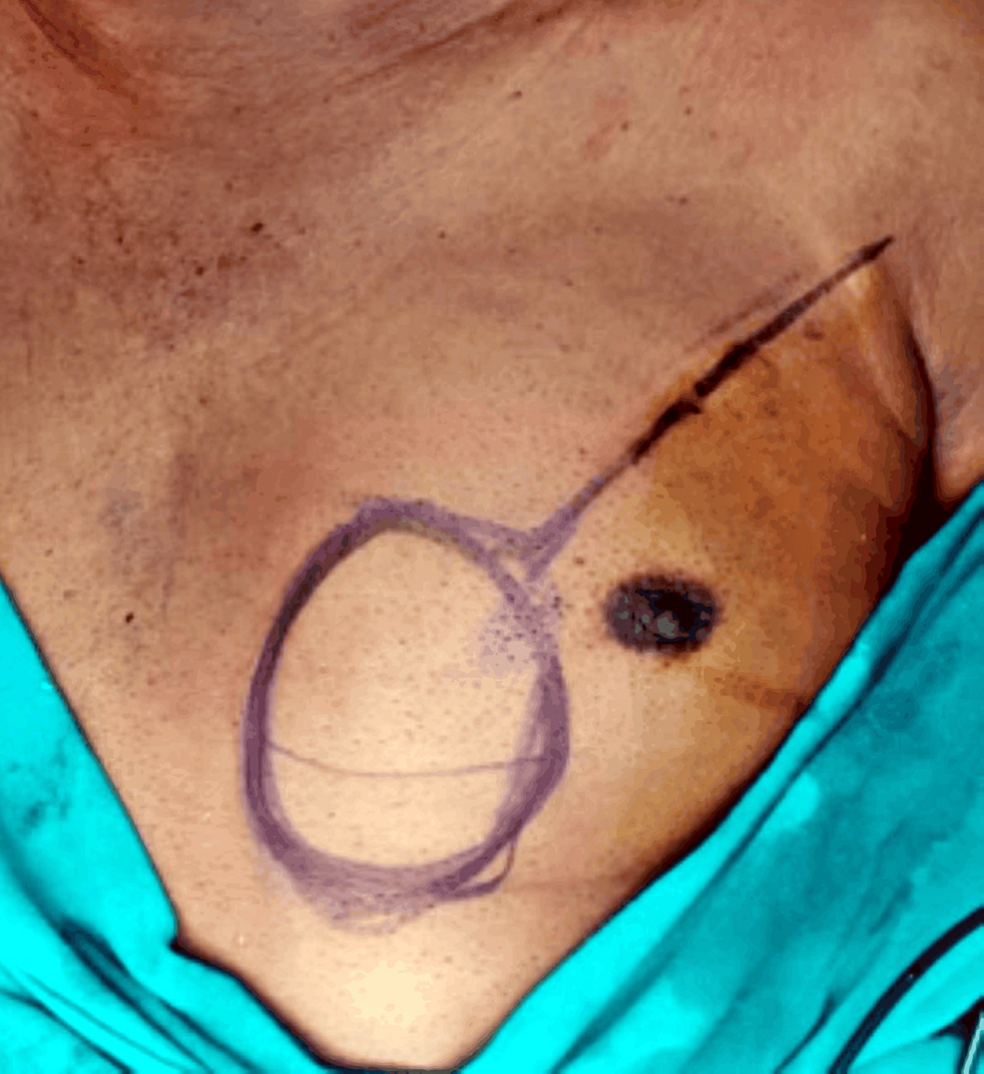
Surface markings were made to determine the vascular pedicle from the shoulder to the xiphisternum

The patient was then subjected to routine laboratory investigation for fitness for surgery under general anesthesia, as shown in Table 1. After preanesthetic fitness and dual informed consent, the patient was posted for surgery. Composite resection of the lesion over the lower left gingivobuccal sulcus with segmental mandibulectomy from the left side first molar to the right side lateral incisor was done (Figure 5). A modified radical neck dissection of the left side was carried out (Figure 6). The defect was reconstructed with the PMMC (Figure 7). To do so, the surface markings were made to determine the vascular pedicle from the shoulder to the xiphisternum (Figure 8). A line was drawn from the clavicle, joining the first line. The skin paddle was drawn over the location of the pectoralis major muscle in such a way that the length of the superior edge of the skin paddle and the inferior aspect of the clavicle matched the size of the intraoral defect and the inferior border of the clavicle to ascertain the length of the pedicle. The incision was taken over the marked skin paddle and extended from the superolateral aspect of the skin paddle vertically until the axillary region. The incision was deepened to the subcutaneous tissue and fat until the pectoralis major muscle was reached. The demarcation of the pectoralis major muscle from the pectoralis minor muscle was ascertained laterally. From the inferior aspect of the skin paddle, the dissection was carried out until the full thickness of the muscle, and the muscle was separated from the underlying rib, dissecting it from the pectoralis minor muscle up until the inferior border of the clavicle. Medially, the flap was separated from the sternum. While doing so, the intermammary artery perforator had to be sacrificed medially. The flap was tunneled over the clavicle and adapted to the defect intraorally. The suturing of the harvested flap was done with a resorbable 2-0 polyglactin suture. After placing a negative suction drain, the donor site suturing was done in layers. The subcutaneous tissue and fat were sutured with a resorbable 2-0 polyglactin suture, and the skin was sutured with a 3-0 polyamide suture. The negative suction drain from the donor site was removed in five days.

**Table 1:** All the laboratory investigation values with their normal reference values

Investigations	Normal values	Patient values	
Hemoglobin count	14-18 g/dL	13.2 g/dL	
Total WBC count	5,000-10,000 cells/μL	10,900 cells/μL	
Mean corpuscular hemoglobin concentration	32-36 g/dL	33.5 g/dL	
Mean corpuscular volume	80-100 fL	87.6 fL	
Mean corpuscular hemoglobin	27-31 pg	29.4 pg	
Total RBC count	3.7-6.1 cells/μL	4.5 cells/μL	
Total platelet count	1.35-3.17 cells/μL	2.07 cells/μL	
Creatinine	1.0 mg/dL	0.9-1.5 mg/dL	
Sodium	137 mEq/L	133-146 mEq/L	
Potassium	4.2 mEq/L	3.5-5.4 mEq/L	
Total bilirubin	0.5 mg/dL	0.2-1.2 mg/dL	
Total protein	7 g/dL	6-8 g/dL	

**Figure 9:**
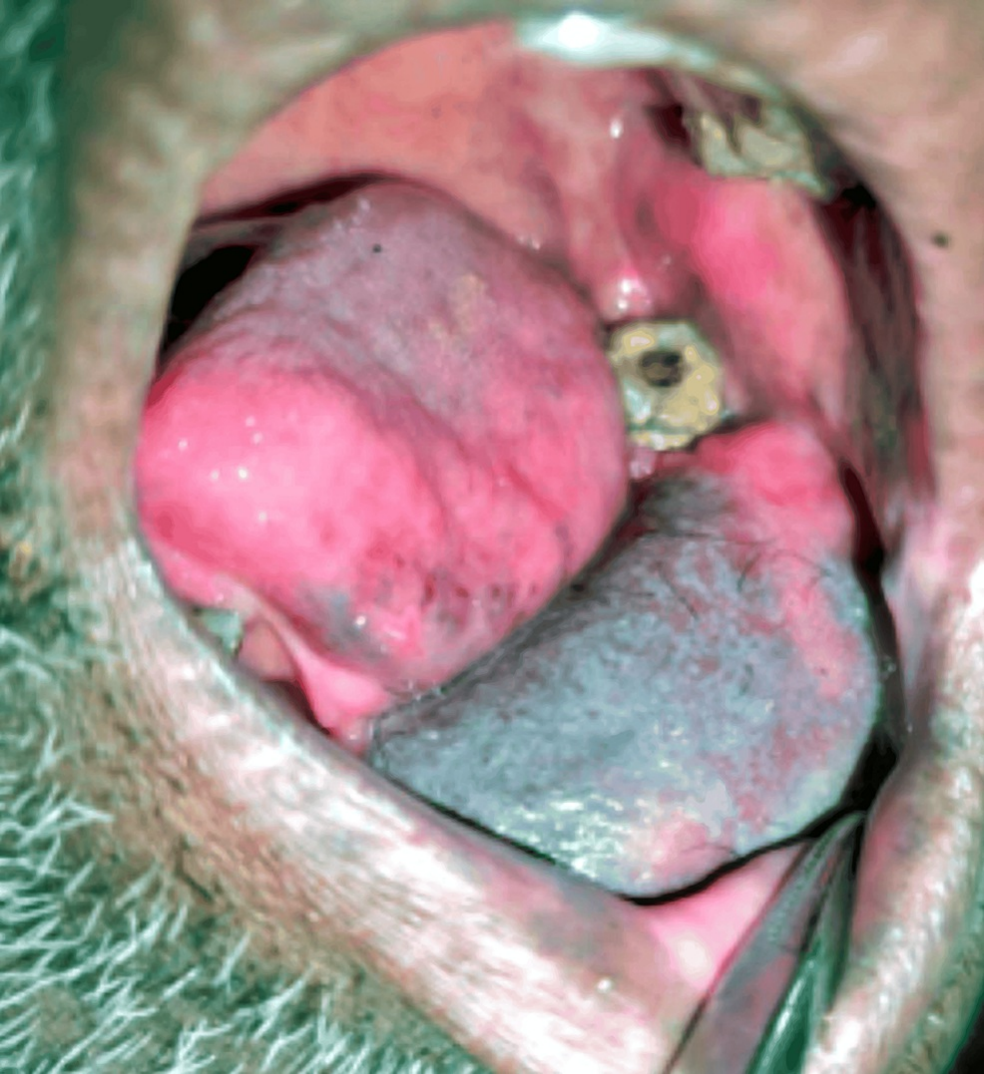
Postoperative treatment intraorally after one-year follow-up

**Figure 10:**
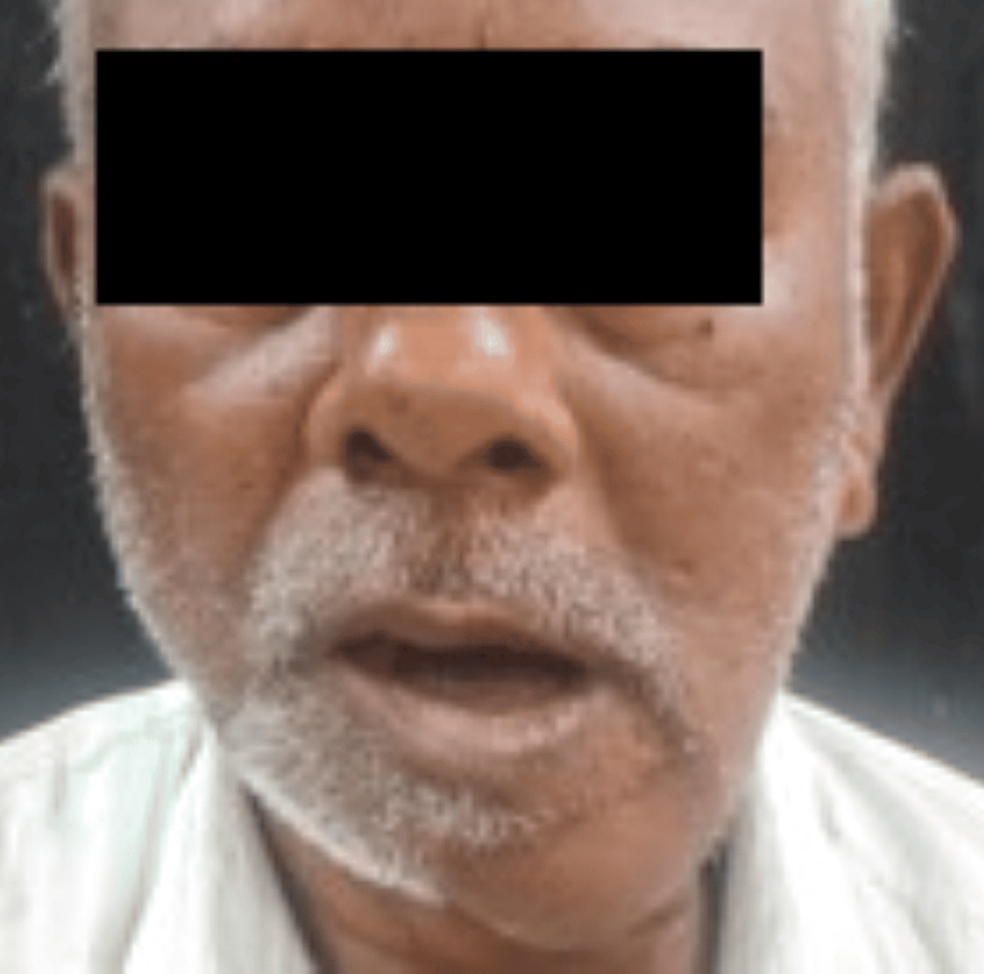
Postoperative frontal view of the patient after one-year follow-up

Postoperative medications are given according to the hospital policy protocol. The surgical specimen histopathological report (HPR) was obtained. Based on the surgical HPR, adjuvant chemoradiotherapy was advised by a multidisciplinary tumor board discussion panel. The patient had 35 radiation therapy sessions with a total of 60 grays and received five cycles of chemotherapy with 50 mg cisplatin injections. The patient was recalled after one year for follow-up, and during follow-up, the patient's physical condition was healthy with adequate mouth opening, the surgical site was healthy, and the healing was satisfactory (Figures 9, 10). There was satisfactory progress after surgery with no evident complications.

## Discussion

Oral cancer presents as an uncontrolled growth of cells that invade and cause the destruction of adjacent structures. It is primarily caused by chronic tobacco use in smoked and smokeless forms. In India, the habit of eating smokeless tobacco is prevalent. A habituated individual keeps the mixture of tobacco and lime in the vestibule. The coarseness of the tobacco causes chronic irritation to the adjacent mucosa. Moreover, the use of lime induces chemical damage [7]. Chronic use of this leads to the development of premalignant lesions. Continuing the habit even through these lesions transforms the lesion into malignant. The local spread of OSCC occurs by infiltration into adjacent tissue. It invades the bone through the alveolar process in dentate patients or the dental pores in edentulous patients. It spreads faster through the cancellous bone than through the cortical bone. It also spreads through the inferior alveolar nerve canal [8].

Oral cancer is a debilitating disease that has low survival rates with the progression of the disease. However, the overall survival rate of patients with SCC of the oral cavity has increased over the last 10 years, primarily due to advances in diagnosis, early detection, treatment of metastatic nodes, and routine follow-up calls. The treatment modalities for OSCC are surgery, chemotherapy, and radiation therapy. The treatment choice depends on the tumor stage, nutritional status of the patient, associated disease, and distant metastasis [9]. However, surgery is the main treatment in these cases. Surgical management involves the excision of the tumor mass in toto along with 1 cm of margin [10]. Treatment of oral cancer depends on the stage of cancer at the time of initial diagnosis, and the stages are determined with the help of the tumor, node, metastasis staging [11]. Many aspects are taken into account while choosing a course of therapy; it is important to customize the plan to each patient's unique needs while considering both the individual's standard of living and the chance of survivability. Single-modal treatment, such as surgery or radiotherapy, is done in early-stage oral cancers (stages I and II).

In cases of involvement of the mandibular bone, mandibulectomy has to be carried out. Segmental mandibulectomy is the removal of the segment of the mandible in toto [12]. It is indicated in cases where there is severe damage to the adjacent mandibular bone, a history of radiotherapy, an edentulous atrophic mandible, paramandibular spread, and an inability to preserve 1 cm of vertical height to minimize the risk of pathological fracture. Marginal mandibulectomy is the removal of a portion of the mandible while maintaining 1 cm of intact bony margin. It is done to obtain adequate margins when the tumor is closely abutting the mandible. It is also indicated in cases with limited periosteal invasion or very superficial bony erosion. In comparison to individuals who received segmental mandibulectomy (70.5%), those who underwent marginal mandibulectomy (33.3%) performed worse when histopathologically identified bone penetration was scrutinized [13].

Reconstruction of the surgical defect after resection of the lesion is aimed at restoring the integrity of the surgical site, retaining its function, and maintaining its form. Following the reconstructive ladder, the ideal reconstruction for composite defects of head and neck cancers is free flaps, as they restore the bulk of the tissue while bringing in its own blood supply. In our case, the ideal reconstruction options were a free fibula flap or a deep circumflex iliac artery (DCIA) flap [14]. However, the DCIA flap has many drawbacks of its own, like the chances of herniation, donor site morbidity, gait disturbances, and lateral cutaneous femoral nerve damage [15]. A free fibula flap is the ideal reconstruction in our case, as it provides the bulk and an adequate amount of bone for reconstruction [16]. Being a farmer by profession, our patient was unwilling to undergo free flap surgery. Hence, we resorted to the PMMC for reconstruction.

The PMMC flap is mainly used in head and neck reconstruction as a salvage flap. However, it has proven to be a workhouse for reconstruction in oral cancer cases. It was first used as an axial pattern flap in head and neck reconstruction [17]. It is based on the pectoral branch of the thoracoacromial artery, which is a branch of the axillary artery. It is a versatile flap and has aided in the reconstruction of almost all the defects of oral cancer. It provides a good length of pedicle that is tunneled over the clavicle and adapted over the defect. It can also provide an extended reach by sacrificing the lateral thoracoacromial artery perforator. It can also be used for defects in which the skin has been sacrificed by bipaddling of the flap [18].

The reconstruction of complex maxillofacial defects requiring both mucosal lining and skin surfacing poses challenges in a single-stage procedure. We present a case in which advanced oral cavity cancer ablative surgery was performed, and reconstruction was carried out. In this case, the flap was likely utilized to cover both intra- and extraoral defects. Using a bipedally pedicled approach means that the flap was anchored or attached at two points, typically to blood vessels or other structures, to ensure adequate blood supply and viability of the tissue. The donor site defect was closed in a single stage. There was minimal facial deformity, good lip competence, and improved function.

On follow-up after a year, the gentleman is healthy and disease-free, with adequate mouth opening, proper lip competency, and intact function. Hence, we can conclude that the bipaddled PMMC may stand out as a modification that has proven highly effective in managing challenging full-thickness defects in head and neck surgery, notably following the excision of oral cancers.

## Conclusions

Reconstruction of the ablative defects can significantly impact the patient's quality of life after surgery. The oral seal has not been properly made, resulting in the patient complaining of drooling, very poor esthetics, and social embarrassment. This case is an example of the challenges faced in diagnosing and treating advanced stages of oral carcinoma, especially in cases of local and regional comorbidities. Furthermore, this case depicts the need to improve further treatment and outcomes and reduce treatment-related complications. This case also highlights the importance of early detection, prompt intervention of the defect with the best reconstructive modality, and regular follow-up with the patient.
